# 3 dimensional modelling of early human brain development using optical projection tomography

**DOI:** 10.1186/1471-2202-5-27

**Published:** 2004-08-06

**Authors:** Janet Kerwin, Mark Scott, James Sharpe, Luis Puelles, Stephen C Robson, Margaret Martínez-de-la-Torre, Jose Luis Ferran, Guangjie Feng, Richard Baldock, Tom Strachan, Duncan Davidson, Susan Lindsay

**Affiliations:** 1 Institute of Human Genetics, University of Newcastle upon Tyne, International Centre for Life, Central Parkway, Newcastle upon Tyne, NE1 3BZ, UK; 2 Medical Research Council Human Genetics Unit, Western General Hospital, Edinburgh, EH4 2XU, UK; 3 Department of Human Anatomy and Psychobiology, University of Murcia, Spain; 4 School of Surgical & Reproductive Sciences, University of Newcastle upon Tyne, Newcastle upon Tyne NE1 4LP, UK

## Abstract

**Background:**

As development proceeds the human embryo attains an ever more complex three dimensional (3D) structure. Analyzing the gene expression patterns that underlie these changes and interpreting their significance depends on identifying the anatomical structures to which they map and following these patterns in developing 3D structures over time. The difficulty of this task greatly increases as more gene expression patterns are added, particularly in organs with complex 3D structures such as the brain. Optical Projection Tomography (OPT) is a new technology which has been developed for rapidly generating digital 3D models of intact specimens. We have assessed the resolution of unstained neuronal structures within a Carnegie Stage (CS)17 OPT model and tested its use as a framework onto which anatomical structures can be defined and gene expression data mapped.

**Results:**

Resolution of the OPT models was assessed by comparison of digital sections with physical sections stained, either with haematoxylin and eosin (H&E) or by immunocytochemistry for GAP43 or PAX6, to identify specific anatomical features. Despite the 3D models being of unstained tissue, peripheral nervous system structures from the trigeminal ganglion (~300 μm by ~150 μm) to the rootlets of cranial nerve XII (~20 μm in diameter) were clearly identifiable, as were structures in the developing neural tube such as the zona limitans intrathalamica (core is ~30 μm thick). Fourteen anatomical domains have been identified and visualised within the CS17 model. Two 3D gene expression domains, known to be defined by Pax6 expression in the mouse, were clearly visible when PAX6 data from 2D sections were mapped to the CS17 model. The feasibility of applying the OPT technology to all stages from CS12 to CS23, which encompasses the major period of organogenesis for the human developing central nervous system, was successfully demonstrated.

**Conclusion:**

In the CS17 model considerable detail is visible within the developing nervous system at a minimum resolution of ~20 μm and 3D anatomical and gene expression domains can be defined and visualised successfully. The OPT models and accompanying technologies for manipulating them provide a powerful approach to visualising and analysing gene expression and morphology during early human brain development.

## Background

Brain development, particularly in human, involves complex changes in shape and structure over time. During a period of approximately 4 weeks (from 26 to 56 days of development; Carnegie stages CS12 to CS23) the major subregions of the human brain are established and development proceeds from a simple neuroepithelial tube to a highly complex three dimensional (3D) structure [1]. For many years it has been recognized that three dimensional models are an important aid to interpreting these developmental changes. In the past, these have been based on labour intensive methods for reconstructing representations of physical sections (e.g. Born reconstructions [2]) or, more recently, on computer-based methods, although these are still labour intensive [3]. Non-invasive techniques have also been used and these have advantages of speed and lack of sectioning artifacts and, for example with MRI, the ability to generate in vivo images and good quality images for larger specimens [4]. Recently, a new, rapid and non-invasive 3D modelling method, Optical Projection Tomography (OPT; [5,6]), has become available, and we have used this method to generate computer-based 3D models from intact early human developmental specimens. OPT has the advantage over MRI in that detailed models can be produced from small samples. With MRI, low signal-to noise ratios make it more difficult to obtain high quality data from embryos younger than CS17 [7]. MRI is likely to be useful for specimens larger than CS23 where the size of the specimen and the density of the tissue are too great to allow penetration of the light.

The OPT models are visualised and manipulated using MAPaint, a suite of software programmes developed as part of the Edinburgh Mouse Atlas Project ([8-10]). The software allows any OPT model to be digitally sectioned in any plane and several different planes can be viewed simultaneously. These planes can be selected at any arbitrary viewing orientation and position through the model. In addition anatomical regions can be defined and manually "painted", allowing the user to interactively assess developing anatomy.

OPT reconstructions were made of human embryos for a number of different stages of development. In all cases, reconstructions were made from autofluorescent imaging – in other words, the intrinsic fluorescence from the fixed specimens was used as the basis for the histological contrast seen in the 3D model. One of the models (CS17; approx. 41 days of development) was examined in detail in order to test the resolving power of the OPT technology on these unstained embryos, in relation to structures in the developing nervous system and to determine the feasibility of using the model as a framework for mapping gene expression patterns. Digital OPT sections were compared with corresponding histological sections stained in 3 different ways: standard haematoxylin and eosin stain to visualise cell nuclei, cytoplasm and connective tissue [11] and immunocytochemistry to detect GAP43 or PAX6 expression. H&E staining distinguishes amongst the ventricular, intermediate and mantle layers in the central nervous system and cranial nerves and ganglia are clearly identifiable. Growth-associated-protein 43 (GAP43) is expressed in growing dendrites and axons [12] and is expressed in the peripheral nervous system and developing tracts of the central nervous system. A number of genes have been identified, particularly in mouse and chick, that are involved in the specification of different brain regions (e.g. reviewed in [13] and [14]). Such gene expression patterns can be used to identify specific brain regions and compare their relative extent in different species (reviewed in [15] and [16]). PAX6 expression is well characterized as defining several regions and boundaries in the developing mouse brain [17,18] and [19]. The effectiveness of using 2D section data to generate 3D expression domains was tested by examining two boundary regions in the CS17 model, the zona limitans intrathalamica (between the dorsal and ventral thalamus) and the midbrain-diencephalon boundary.

## Results and Discussion

**Figure 1:**
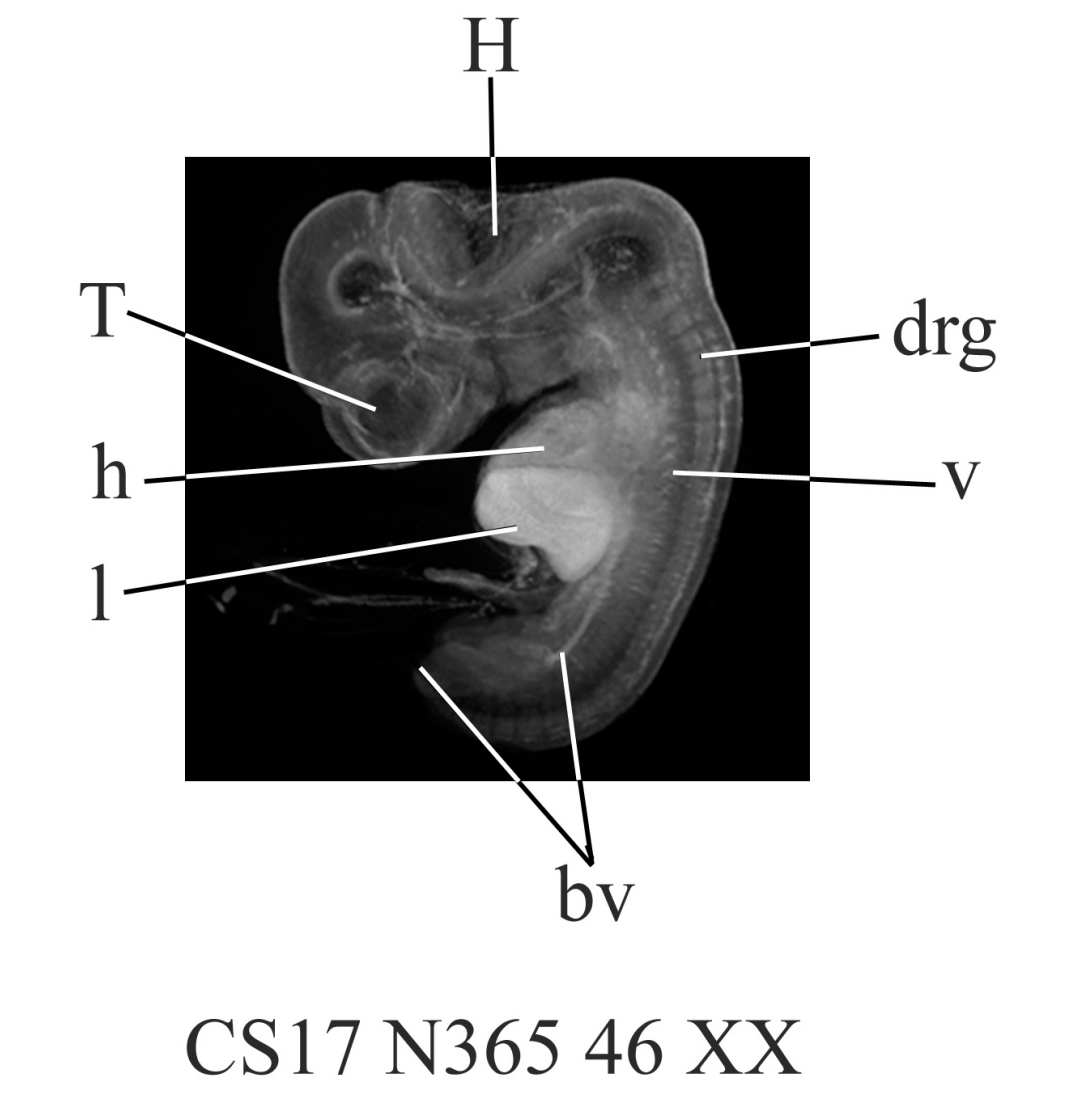
**CS17 OPT model **(a). still shot from movie of 3D OPT model of a CS17 human embryo (approximately 41 days of development). bv, blood vessel; drg, dorsal root ganglion; h, heart; H, hindbrain; l, liver; T, telencephalon; v, vertebrae. (b; Additional file 1) Mpeg movie of 3D CS17 OPT model.

An OPT model was generated from an intact, unstained embryo which had been staged according to the Carnegie staging protocol [20] modified for use with individual embryos rather than in comparisons of many embryos simultaneously [21,22]. The embryo was staged as CS17, which is approximately 41 days of development. The developing central nervous system (CNS) is clearly visible even in the external view of the CS17 model (Figure 1 and the accompanying movie, additional file 1 1). Differences in autofluorescence within the CNS and among different organs are also apparent. There is very little detail in the developing liver, for example, compared to the CNS. Blood vessels, dorsal root ganglia and developing vertebrae are clearly visible.

**Figure 2:**
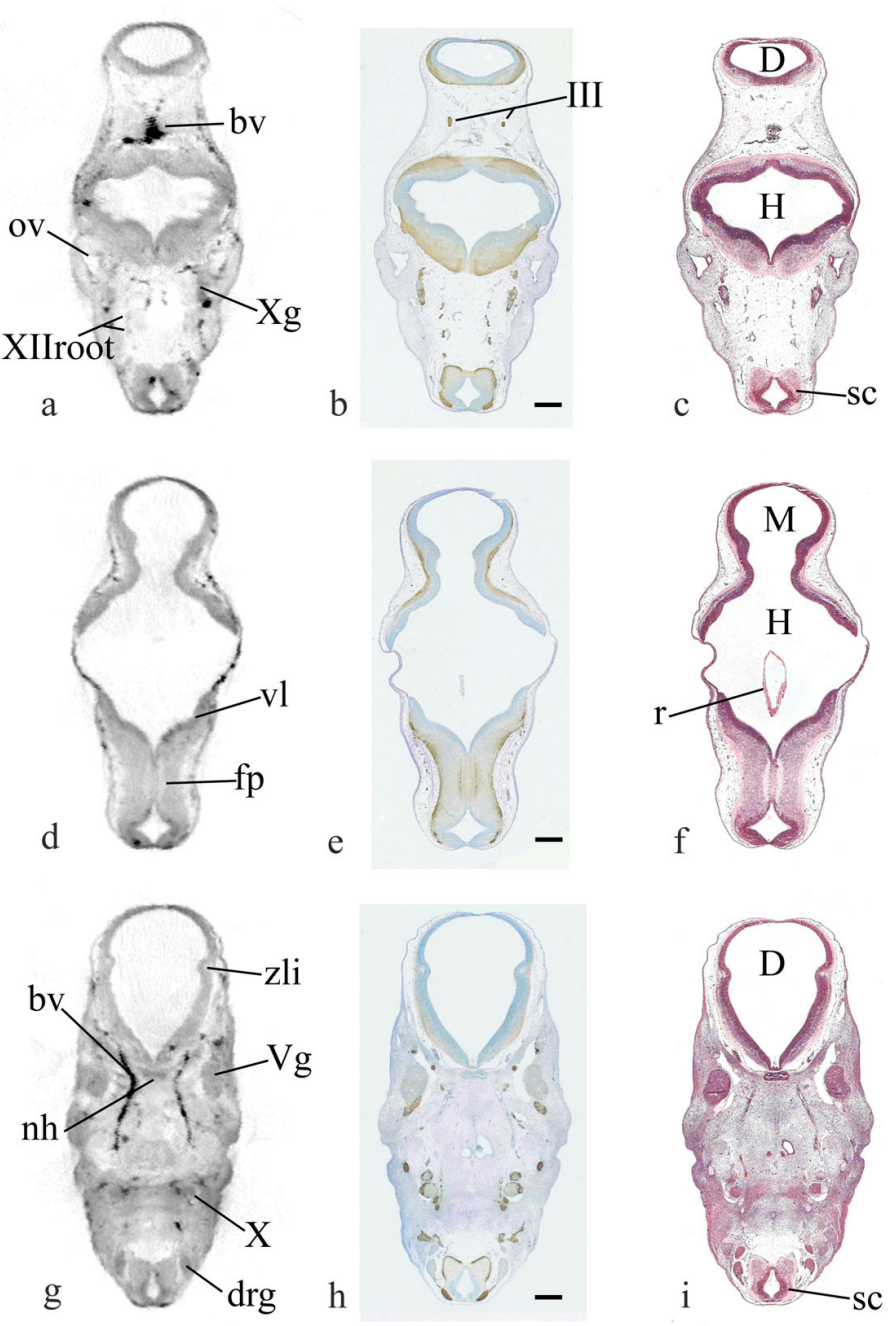
**Comparison of digital OPT sections with histology sections from the same embryo **Digital OPT sections of the CS17 model (a, d and g), viewed using MAPaint software, compared with sections stained using antibodies against GAP43 (b, e and h), and Haematoxylin and Eosin stained sections (c, f and i). In b, e and h, expression is demonstrated by the brown chromagen. Structures of 20 μm in diameter (for example the hypoglossal rootlets) are clearly identifiable, as are the differences amongst a variety of developing tissues. bv, blood vessel; D, diencephalon; drg, dorsal root ganglion (~150 μm); fp, floor plate; H, hindbrain; III, oculomotor nerve; M, midbrain; nh, neurohypophysis (~200 μm by ~50 μm); ov, otic vesicle; r, collapsed roof of 4th ventricle; sc, spinal cord; Vg, trigeminal ganglion (~300 μm by ~150 μm); vl, ventricular layer; X, vagus nerve; Xg, vagus ganglion; XIIroot, hypoglossal rootlets (~20 μm); zli; zona limitans intrathalamica (~100 μm, core is ~30 μm). Scale bars = 200 μm

Following OPT the embryo was embedded in paraffin wax and sectioned using standard methods and then every fifth section was either stained with haematoxylin and eosin or immunocytochemically with antibodies against GAP43 or PAX6. The actual plane of sectioning was identified in the OPT model by manipulating the model in MAPaint. This permitted a matched series of digital and physical sections to be compared (examples are shown in Figure 2) in order to assess the resolution of internal structures within the CS17 OPT model. Different features are highlighted by the different staining techniques and some examples of these are also shown in Figure 2. The cranial nerve ganglia stain partially with GAP43 (e.g., Xg) and in the H&E sections (e.g., Vg) and are clearly visible in the OPT model (Figure 2a and 2g). Cranial nerves also stain for GAP43 (e.g., III) but are less visible in both the H&E section and the OPT model. Surprisingly the rootlets of cranial nerve XII do show up clearly in the OPT model (Figure 2a). In the CNS, the ventricular layer can be clearly distinguished as more darkly staining in the H&E sections and it is also visible as a darker layer in the OPT model (Figure 2a,2d and 2g). The core of the zona limitans intrathalamica (zli) is seen as a pale region in all three sections (2g,2h,2i). The GAP43 stained fibres in the floor of the hindbrain (2e) show clearly as a pale area in the OPT model (2d). As was visible in figure 1, blood vessels and dorsal root ganglia show up very clearly in the OPT model (2a and 2g). Fluid filled spaces are also clearly visible in the OPT model, such as the diencephalic, midbrain and hindbrain ventricles and the otic vesicle (2a and 2d). Some artefactual changes in shape seen in the physical sections (for example, a collapse of the fourth ventricle roof [r in fig. 2f]) are not present in the OPT model (compare 2d and 2f). The size of a variety of structures was measured in the H&E and/or GAP43 stained sections in order to determine the resolution in the CS17 model. When OPT is performed on specimens in which specific cells have been fluorescently-labelled, then these small structures can be detected (unpublished data). However, in cases where the specimen contains no tissue-specific dyes, a resolution of 5 to 10 micron has previously been found [5]. At the magnification used to generate this CS17 model, the smallest clearly measurable structures are the rootlets of the XII cranial nerve which are approximately 20 μm in diameter (Figure 2a).

**Figure 3:**
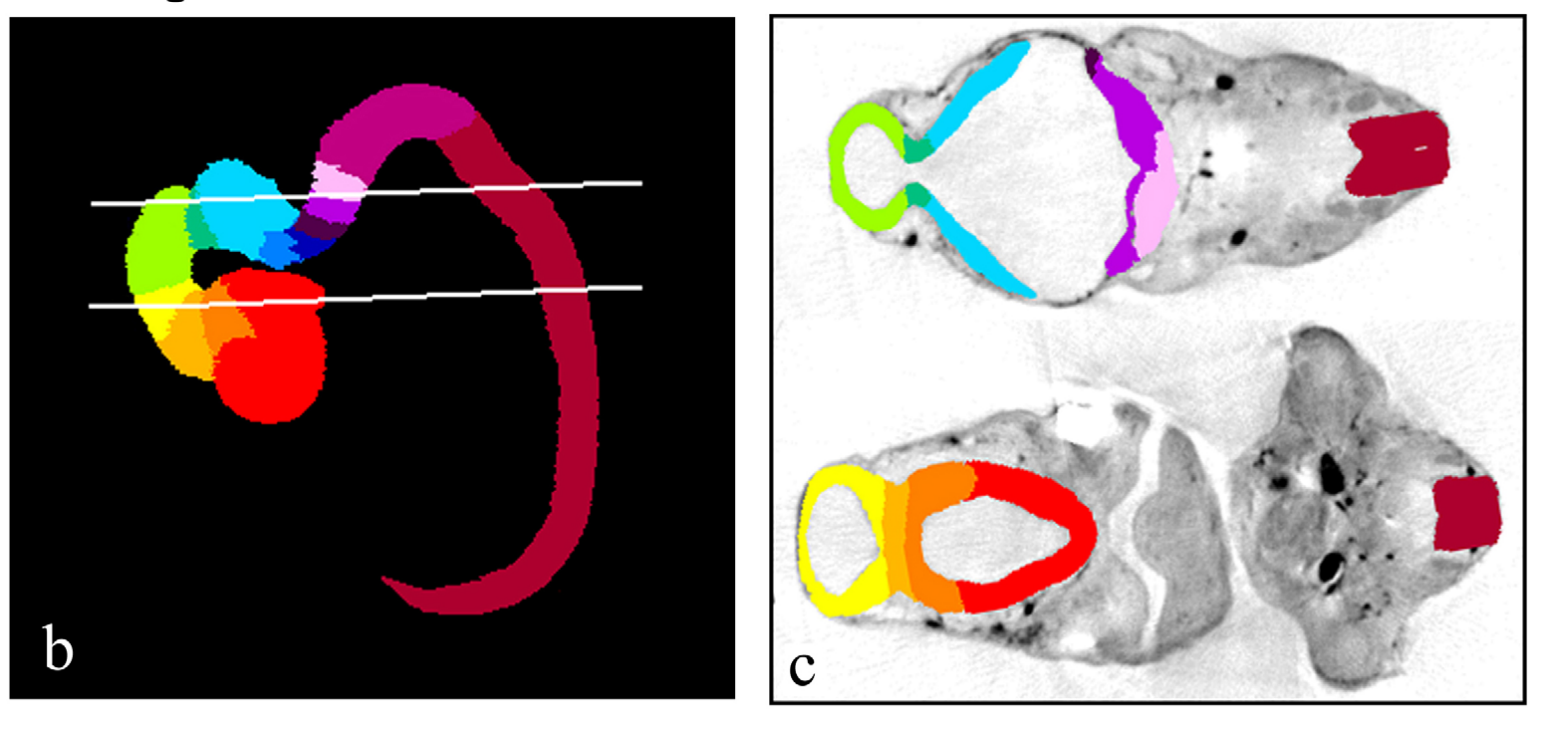
**Painted anatomical domains. **Fourteen regions of the central nervous system in the CS17 specimen have been defined and painted. **Forebrain**, red (secondary prosencephalon), dark orange (prosomere 3 including ventral thalamus), light orange (prosomere 2 including dorsal thalamus) and yellow (prosomere 1 including pretectum); **midbrain**, light green; **hindbrain**; isthmus, dark green; various shades of blue and purple indicate rhombomeres 1–6 and the caudal medulla oblongata; **spinal cord**, dark red. (a; Additional file 2) In the Mpeg movie sagittal and transverse views of the painted model are shown, together with a representation of the 3D domains. The model is first sectioned in the transverse plane. This section plane has been matched to that of the histology sections shown in fig 2. As the section is moved through the model the corresponding position is displayed in the 3D box, and by a line on the sagittal section. The model is then moved through the sagittal plane, and the position shown by a line on the transverse section. A snapshot of the fourteen 3D anatomical domains (b), and two examples of painted sections that intersect several anatomic domains (i.e., are topologically nearly horizontal to the reconstructed transverse boundaries) (c). The position of the two digital transverse sections is indicated by white lines on the 3D view.

MAPaint, a UNIX-based software suite, allows the OPT models to be digitally sectioned in any plane and several planes to be viewed simultaneously. This is illustrated for the CS17 model in Figure 3a (additional file 2) which moves through a series of digital transverse sections with the corresponding position of each section shown on a sagittal section, followed by a series of sagittal sections and the corresponding position on a transverse section. The CS17 model is shown with some painted anatomical domains and Figure 3b shows a snapshot of all fourteen 3D domains with the position of the two transverse sections shown in Figure 3c indicated in white. The anatomical domains were painted in one plane and then checked and refined by corrections introduced in orthogonal planes. The ease of manipulation of the model means that the best digital plane (normally orthogonal to any boundary or set of them) can be selected for each anatomical region being painted. The authors who did these tracings actually found the experience intellectually rewarding, since it amounted to being able to check instantly any difficult point upon a collection of identical specimens sectioned in many different planes. Doubts that often remain unresolved could be resolved very convincingly.

**Figure 4:**
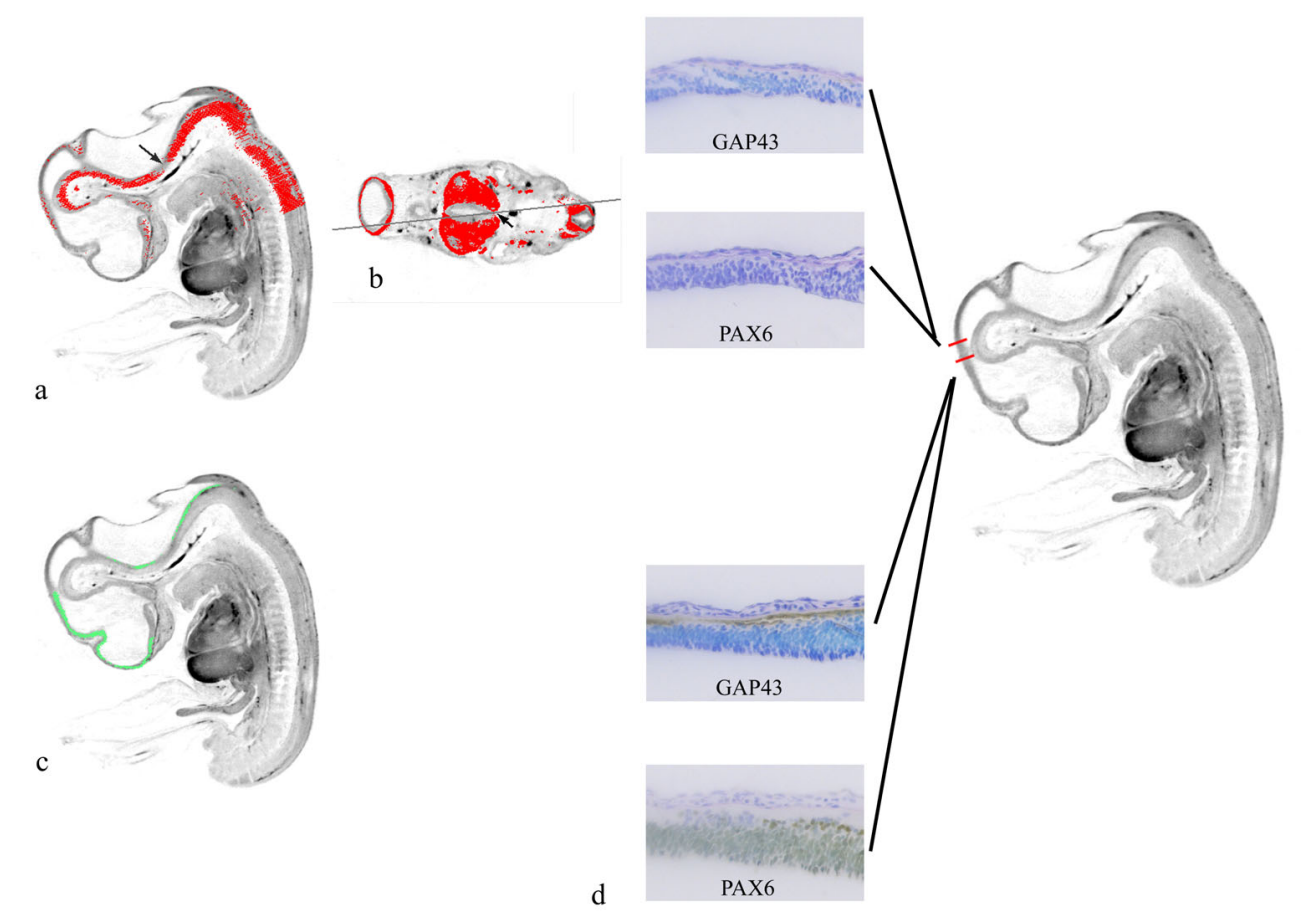
**CS17 OPT model showing 3D gene expression domains. **(a) Digital sagittal section through the CS17 OPT model, with the GAP43 gene expression domain shown in red. The plane of this sagittal section is shown by a line on the corresponding transverse section in (b). The GAP43-negative region in the hindbrain floor plate is shown on both sections by an arrow. (c) The same digital sagittal section, with PAX6 gene expression displayed in green. (d) High power images of GAP43 and PAX6 expression near the diencephalon/midbrain boundary. The upper two panels correspond to the rostral midbrain, where there is no expression of GAP43 or PAX6. The lower two panels correspond to the caudal diencephalon, in the region of the posterior commissure. Here both genes are expressed (brown chromagen).

**Figure 5:**
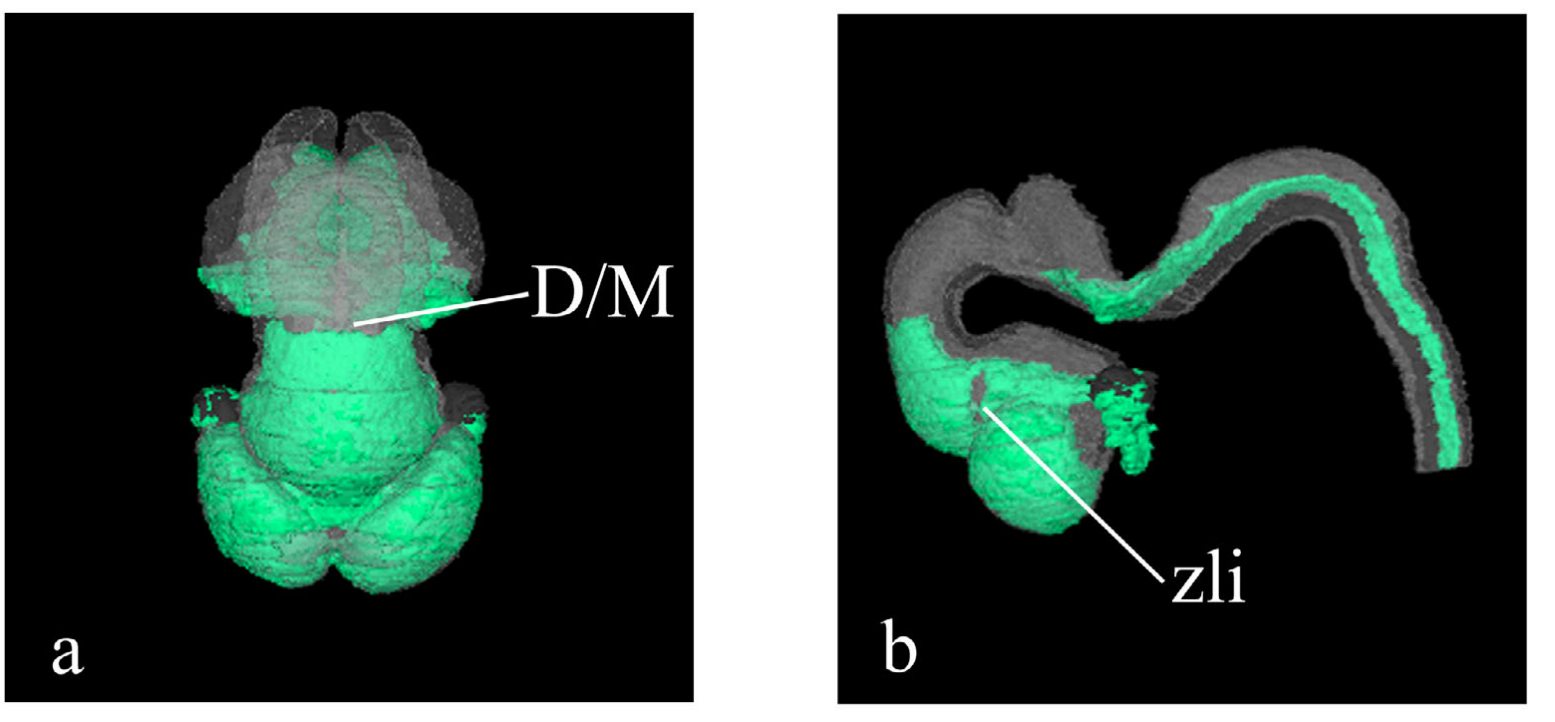
**3 dimensional gene expression domains. **A surface rendered model of the 3D expression pattern of PAX6. Separate gene expression domains in the forebrain and hindbrain are shown in green. For reference the neural tube has been painted pale grey and the eye dark grey. The diencephalon/midbrain (D/M) boundary, the absence of staining in the zona limitans intrathalamica (zli), plus the forebrain alar-basal boundary and the striatopallidal boundary in the basal telencephalon can be seen by viewing the 3D model at various angles (a, frontal and b, lateral). (c; Additional file 3) Mpeg movie of the PAX6 expression domain.

As described in the Methods section, and in the legend to Figure 2, paraffin sections were immunostained with either anti-GAP43 or anti-PAX6 antibodies at approximately 50 μm intervals throughout the head of the CS17 specimen and the data were captured and mapped to the CS17 model. Figure 4a illustrates a sagittal section through the model with digital GAP43 expression (in red) which has been generated from the data thresholded from transverse sections, an example of which is shown in Figure 4b. There is an unexpected apparent region of no GAP43 expression in the hindbrain on the sagittal section (Fig 4a, arrow). However, the ability to relate the sagittal and transverse sections in the model makes clear that the lack of expression is due to the sagittal section being "cut" obliquely and the section is passing through the floor plate region of the hindbrain at that point, where there is no GAP43 expression (arrowed in Figure 4b). A sagittal section with digital PAX6 expression shown in green also illustrates the lack of PAX6 expression in the same floor plate region in the hindbrain (Figure 4c). Comparing Figure 4a and 4c it can be seen that the boundary of the PAX6 expression in the caudal diencephalon is approximately the same boundary as that of GAP43 which stains the posterior commissure, just caudal to which is the boundary between the pretectum, the most caudal region of the developing diencephalon [23,24] and the midbrain. The lack of PAX6 and GAP43 expression in the region just caudal to the posterior commissure is shown (Figure 4d; upper 2 panels) while the expression of both GAP43 and PAX6 in the pretectum is shown in the lower two panels of figure 4d. The 3D PAX6 domain identifies both the diencephalon-midbrain boundary and the negative intrathalamic boundary or zli (as shown in Figure 5a,5b and the accompanying movie (Additional file 3). Additional limits of PAX6 expression visible in Figure 5b correspond to the basal telencephalon (the striatopallidal boundary) and the alar-basal boundary across the entire diencephalon [25,23,24]. Separate areas of PAX6 expression appear in the hindbrain (Figs. 5a,5b)

**Figure 6:**
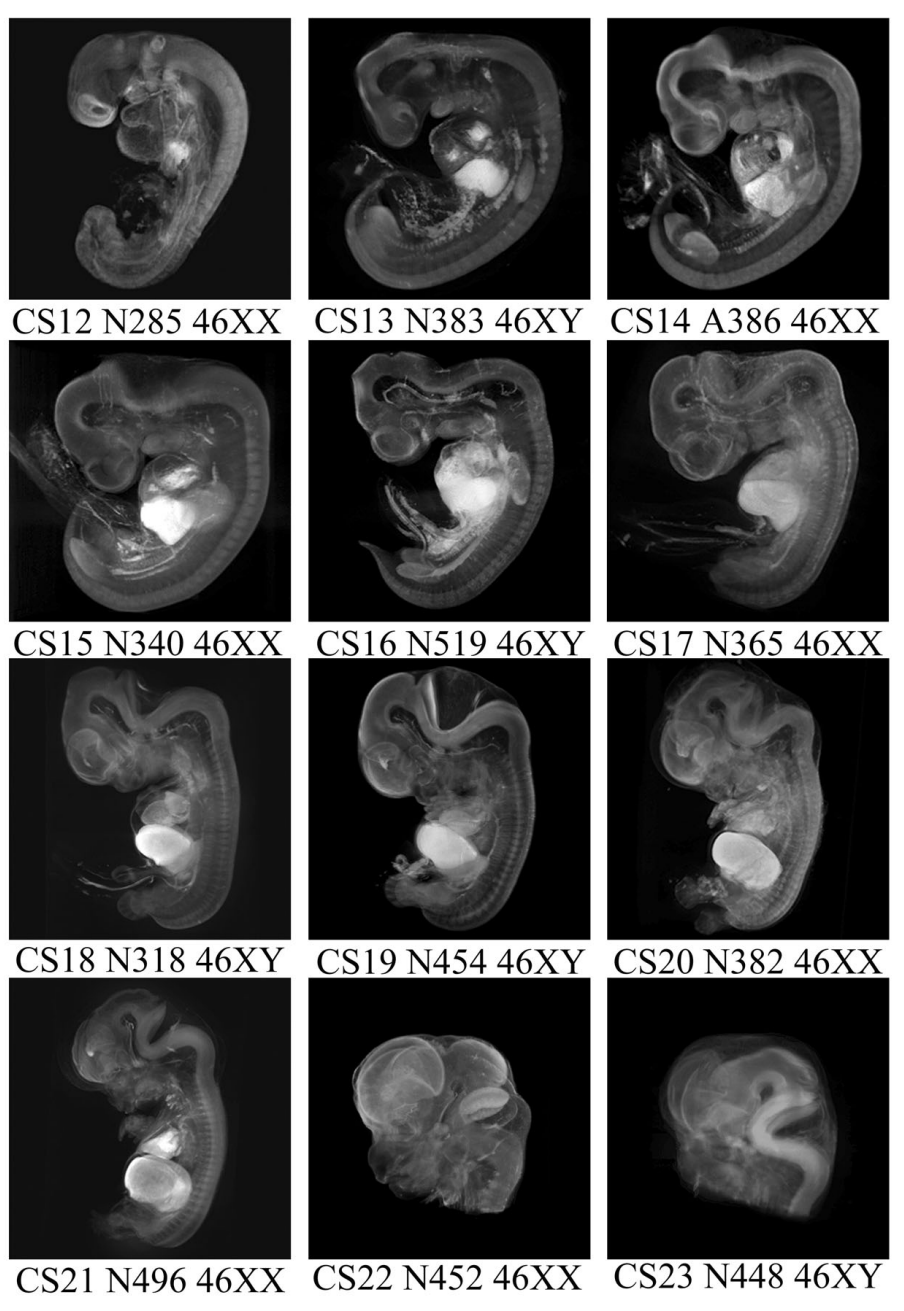
**OPT models of CS12 to CS23. **Still shots of the left lateral side of 3 dimensional OPT models of human embryos spanning the major period of organogenesis (CS12-CS23). The developmental stage (e.g. CS12), specimen number (e.g. N285) and karyotype for each model are given underneath. The movies for all of these models can be viewed at  [27]. The full models for all stages are available on request.

The period from CS12 (approximately 26 days of development) to CS23 (approximately 56 days of development) is important because during this time all the major regions of the developing brain are established [26,1] and most major congenital abnormalities can arise. Major changes take place in the size, shape and complexity of the developing brain during this time and we tested the feasibility of generating OPT models throughout this period. There is a more than 60 fold increase in volume between the CS12 embryo and the CS23 head, however, OPT models have been generated successfully throughout these stages. Figure 6 shows snapshots of models at each of the stages from CS12 to CS23. These are not to scale because each model is generated at the maximum magnification possible, which varies according to the size of the specimen. The changes in shape and complexity are clearly seen even in these static images. We currently have 54 OPT models, including a male and female at each stage CS12-CS20 and CS22. At CS17 we have twelve different OPT models and have assessed their natural variability for 3 specific neurodevelopmental features (development of the choroid plexus in the lateral ventricles, the zli and the floor plate in the hindbrain). At this stage, there was little variation in the features assessed (data not shown). Movies of all these models can be viewed at our website ([27]), which includes a database of gene expression. The models are too large to display fully or to manipulate online, but a selection of the full models are available on CD on request and a new viewer, the Java Atlas Viewer, has been developed which enables the models to be viewed and manipulated on many platforms (Burton, Feng, Hill and Baldock, paper in preparation). See additional files 4 and 5 for the request form and Academic use licence agreement.

## Conclusions

Many structures within the developing nervous system can be identified in the CS17 OPT model with a minimum defined resolution of approximately 20 μm. The CS17 model also acted as a framework onto which anatomical domains were easily painted and gene expression patterns mapped. OPT models have been successfully generated from CS12 to CS23, and these models will provide a means of analyzing and relating changes in anatomy and gene expression both within individual developmental stages and across developmental time.

In the long-term, our aim is to link the 3D models to an anatomical database and embed both of them within a custom-designed gene expression database in order to create an Electronic Atlas of the Developing Human Brain ([27]).

## Methods

### Embryo collection

Human embryos were collected from termination of pregnancy material, with appropriate written consent, approval from the Newcastle and North Tyneside NHS Health Authority Joint Ethics Committee and following national guidelines [28]. Embryos were collected into cold PBS, separated from surrounding tissue and fixed overnight in 4% paraformaldehyde at 4°C before short-term storage at 4°C in 70 % ethanol. Placental tissue was sampled for karyotype analysis prior to fixation of the embryo tissue.

### OPT

Intact specimens were rehydrated through a graded series of ethanol and embedded in a block of 1% low melting point agarose. They were then dehydrated through a graded series of methanol before being cleared using a mixture of benzyl alcohol and benzyl benzoate. 400 digital images were then captured while the now almost transparent specimens were rotated in a full circle, with 0.9° steps between each image. The signal corresponded to the weak autofluorescence originating from the paraformaldehyde-fixed tissue and was detected using a wideband FITC filter with excitation at 465–500 nm and emission from 515–560 nm. The images were then assembled to recreate the 3D shape of the embryo, using modified tomography algorithms [5].

### Post OPT processing and histology

After OPT scanning the CS17 embryo N365 was rehydrated through a graded series of methanol and was then removed from the agarose block by incubation in a 0.29 M sucrose solution at 55°C. It was then processed for paraffin wax embedding and 5 μm microtome sections were cut. Every 5^th ^section was stained with haematoxylin and eosin, following standard procedures.

### Immunohistochemistry

The remaining N365 sections were used for immunohistochemistry. Alternate one-in-five section series were stained with antibodies against GAP43 (GAP-7B10, Sigma), or PAX6 (PRB-278P, Covance) using standard techniques. The reaction was visualised with diamino benzidine and the sections lightly counterstained with toluidine blue.

### Gene Expression Mapping

Images of the stained sections were captured through a ×2.5 objective (as viewed down the microscope at 25× magnification) using the Zeiss Axiovision system. For the PAX6 and GAP43 data, a modified warping interface of the MAPaint software was used to match each stained, physical section to the corresponding digital OPT section. Correspondences between the physical (source) and digital (target) images were identified and manually tie-pointed. The source image was then transformed to the shape of the target section, and the image transformation saved. The interface uses interactive thresholding to extract the expression signal from the source image and then applies the image transformation to map this signal into the space of the 3D OPT model. This was carried out for approximately 190 sections through the head of the CS17 embryo until the full 3D expression pattern was built up for each of the GAP43 and PAX6 data sets.

## Authors' contributions

JK reconstructed the OPT data, carried out the immunocytochemistry, mapped the GAP43 gene expression patterns to the CS17 model and drafted the manuscript. MS optimised the OPT methodology for human specimens, scanned the embryos, carried out the sectioning and mapped the PAX6 gene expression. JS invented the OPT technique and established the methodology in Newcastle. LP, MdlT and JLF identified and painted anatomical regions of the CS17 neural tube. GF developed the Java Atlas Viewer. RB designed/authored the MAPaint software and with DD heads the Edinburgh Mouse Atlas Project. TS, SCR and SL co-ordinate and oversee the Human Developmental Biology Resource which provided the human material. SL and TS conceived of the study and participated in its design and coordination. All coauthors participated in drafting or refining the text.

## List of abbreviations

3D-three dimensional

4D-four dimensional

bv-blood vessel

CNS-central nervous system

CS-Carnegie Stage

D-diencephalon

D/M-midbrain/ diencephalon boundary

drg-dorsal root ganglion

EADHB-Electronic Atlas of the Developing Human Brain

fp-floor plate

GAP43-Growth Associated Protein 43

h-heart

H&E-haematoxylin and eosin

H-hindbrain

III-oculomotor nerve

l-liver

M-midbrain

nh-neurohypophysis

OPT-optical projection tomography

ov-otic vesicle

r-collapsed roof of 4^th ^ventricle

sc-spinal cord

T-telencephalon

v-developing vertebrae

Vg-trigeminal ganglion

X-Vagus nerve

Xg-Vagus ganglion

XIIroot-hypoglossal rootlets

Zli-zona limitans intrathalamica

## Supplementary Material

Additional File 1 CS17 OPT model.mpg. Mpeg movie of 3D CS17 OPT model. Click here for file

Additional File 2 CS17 painted anatomy.mpg. Mpeg movie of anatomical domains painted on the CS17 model. Sagittal and transverse views of the painted model are shown, together with a representation of the 3D domains. Click here for file

Additional File 3 PAX6 3D expression.mpg. Mpeg movie of the 3D PAX6 expression domain. Click here for file

Additional File 4 JAtlasViewer request form.pdf Click here for file

Additional File 5 Academic Licence Agreement.pdf Click here for file
