# Supplementary material for: 3 dimensional modelling of early human brain development using optical projection tomography
Source: BMC Neurosci. 2004 Aug 6;5:27. doi: 10.1186/1471-2202-5-27 (PMC514604; doi:10.1186/1471-2202-5-27)
Supplement: Additional File 4 — JAtlasViewer request form.pdf [file 1471-2202-5-27-S4.pdf]

EADHB Model and JAtlasViewer
CD-ROM Request Form

You can request a copy of the CD-Rom by filling out this form and returning it to us at the contact details below. You may send a hard copy to us at the postal address or for convenience you may fill this out as a word document or rich-text-file and e-mail us the form as an attachment. If there are any problems please feel free to contact us at the e-mail address below. Please allow up to 28 days for delivery:

EADHB Models and JAtlasViewer CD-ROM
Institute of Human Genetics
International Centre for Life
Central Parkway
Newcastle upon Tyne
NE1 3BZ
United Kingdom
eadhb@ncl.ac.uk

The CD will be sent as soon as possible.

**Requesters Name:**	
**Institution:**	
**Address:**	
**Phone Number:**	
**Fax Number:**	
**Contact e-mail:**	

I have read and agree to the terms of use located on the EADHB Web Page:

Signed:

Date:
