# Supplementary material for: 3 dimensional modelling of early human brain development using optical projection tomography
Source: BMC Neurosci. 2004 Aug 6;5:27. doi: 10.1186/1471-2202-5-27 (PMC514604; doi:10.1186/1471-2202-5-27)
Supplement: Additional File 5 — Academic Licence Agreement.pdf [file 1471-2202-5-27-S5.pdf]

Academic Use License Agreement

The content of the EADHB CD-ROMs, including text, images, software and anatomical nomenclature is not in the public domain. The CD-ROMs are available free of charge to academic organisations for non-profit research purposes. The licence is for academic organisations only. Commercial organisations wishing to take out a licence should contact the Institute of Human Genetics. The contacts for the CD-ROM are

Dr S Lindsay and Prof. T Strachan
Institute of Human Genetics
International Centre for Life
Central Parkway
Newcastle upon Tyne
NE1 3BZ, UK.
Tel: +44-(0)191-241-8611
Fax: +44-(0)191-241-8666
Email: eadhb@ncl.ac.uk

Please read the terms of use below:

1. The Medical Research Council, UK and the University of Edinburgh, UK hold the copyright for the Software being offered for use on these pages. The Reconstructions and movies are the property of the Institute of Human Genetics, Newcastle and will not be used without prior written consent.

2. The use of the software and the CD-ROMs is granted on the basis that they will be used solely for non-profit academic research purposes and any other use must be by written permission from the Institute of Human Genetics, Newcastle and the MRC Human Genetics Unit, Edinburgh.

3. Any 2D images, movie sequences and anatomical nomenclature contained can be used within publications of the Licensee's organisation provided full and proper reference to the material is made, and that there is no-profit from such publication. For example, images may be used and the text quoted in both paper and electronic publications. If the scientific results of this work derive from an analysis of the data on the CD-ROM directly (as distinct from interpreting new data), then the licensee agrees to contact the Institute of Human Genetics, Newcastle via one or more of the contacts above before publication to obtain permission to use that data in that way.

4. The Licensee agrees to acknowledge the use of the CD-ROM and appropriate software in any document which has input from work using the CD-ROM, including but not limited to published research. The licensee agrees to notify the Institute of Human Genetics via one or more of the contacts above and to supply a copy of the publication, with appropriate modification made forprotection of confidential or proprietary information.

5. THE MEDICAL RESEARCH COUNCIL (MRC), UNIVERSITY OF EDINBURGH, INSTITUTE OF HUMAN GENETICS, NEWCASTLE AND THE UNIVERSITY OF NEWCASTLE MAKE NO REPRESENTATION ABOUT THE SUITABILITY OR ACCURACY OF THIS SOFTWARE OR DATA FOR ANY PURPOSE, AND MAKES NO WARRANTIES, EITHER EXPRESS OR IMPLIED, INCLUDING THE WARRANTIES OF MERCHANTABILITY AND FITNESS FOR A PARTICULAR PURPOSE OR THAT THE USE OF THIS SOFTWARE OR DATA WILL NOT INFRINGE ANY THIRD PARTY PATENTS, COPYRIGHTS, TRADEMARKS OF OTHER RIGHTS. IT IS PROVIDED "AS IS".
